# EIF5A2 enhances stemness of epithelial ovarian cancer cells via a E2F1/KLF4 axis

**DOI:** 10.1186/s13287-021-02256-2

**Published:** 2021-03-16

**Authors:** Kun Wang, Yiyang Wang, Yuanjian Wang, Shujie Liu, Chunyan Wang, Shuo Zhang, Tianli Zhang, Xingsheng Yang

**Affiliations:** 1 Department of Obstetrics and Gynecology, Qilu Hospital of Shandong University, Jinan, Shandong 250012 People’s Republic of China; 2 Affiliated Reproductive Hospital of Shandong University, Jinan, Shandong 250012 People’s Republic of China; 3 West China School of Medicine, Sichuan University, Sichuan, People’s Republic of China

**Keywords:** Ovarian cancer, Cancer stem cells, Chemoresistance, EIF5A2, E2F1, KLF4

## Abstract

**Background:**

Ovarian cancer stem cells (OCSC), endowed with tumor-initiating and self-renewal capacity, would account not only for the tumor growth, the peritoneal metastasis, and the relapse, but also for the acquisition of chemotherapy resistance. Nevertheless, figuring out their phenotypical and functional traits has proven quite challenging, mainly because of the heterogeneity of ovarian cancer. A deeper understanding of OCSC mechanisms will shed light on the development of the disease. Therefore, we aim to explore it for the design of innovative treatment regimens which aim at the eradication of ovarian cancer through the elimination of the CSC component.

**Methods:**

In this study, immunohistochemistry assay and western blot assay were used to detect protein expression in the primary tumor and peritoneal multi-cellular aggregates/spheroids (MCAs/MCSs). OCSCs induced from cell line SKOV3 and HO-8910 were enriched in a serum-free medium (SFM). The effect of EIF5A2 on CSC-like properties was detected by sphere-forming assays, re-differentiation assays, quantitative real-time polymerase chain reaction, western blotting, flow cytometry, cell viability assays, immunofluorescence staining, and in vivo xenograft experiments. RNA-sequencing (RNA-seq) was used to reveal the mechanism by which EIF5A2 positively modulates the stem-like properties of ovarian cancer cells.

**Results:**

Expression of EIF5A2 was significantly higher in peritoneal MCAs/MCSs compared to matched primary tumors, and EIF5A2 was also unregulated in ovarian cancer cell line-derived spheroids. Knockdown of EIF5A2 reduced the expression of the stem-related markers (ALDH1A1 and OCT-4), inhibited self-renewal ability, improved the sensitivity to chemotherapeutic drugs, and inhibited tumorigenesis in vivo. Mechanistic studies revealed that EIF5A2 knockdown reduced the expression of KLF4, which could partially rescue stem-like properties abolished by EIF5A2 knockdown or strengthened by EIF5A2 overexpression, through the transcription factor E2F1, which directly bind to KLF4 promoter.

**Conclusion:**

Our results imply that EIF5A2 positively regulates stemness in ovarian cancer cells via E2F1/KLF4 pathway and may serve as a potential target in CSCs-targeted therapy for ovarian cancer.

**Supplementary Information:**

The online version contains supplementary material available at 10.1186/s13287-021-02256-2.

## Background

Global statistics of ovarian cancer showed that there are 239,000 new cases (3.6% of all cancer cases) and 152,000 deaths each year (4.3% of all cancer deaths), making it the seventh most common cancer, the eighth most common cause of female cancer death, and the second most common cause of gynecologic cancer death following by cancer of the cervix uteri [1]. Studies from UK and US registries estimated that one in six women die within the first 90 days of diagnosis, accompanied by the mortality-to-incidence ratio in ovarian cancer over 0.6, caused by presentation with advanced stage and challenges to effective treatment, which are due in part to the lack of specific, early warning symptoms or signs and lack of effective early screening options [2]. Epithelial ovarian cancer (EOC) accounts for over 95% of ovarian malignant tumor [3, 4]. Given the high incidence of EOC compared with other ovarian cancer histologies, this study will focus on the molecule mechanism of EOC development. For the treatment of EOC, cytoreductive surgery combined with platinum-based chemotherapy has remained the mainstay of therapy for decades [5]. But as the treatment progressed, platinum resistance and relapse are the most important cause of the high mortality rate of ovarian cancer [6]. Intraperitoneal implantation metastasis is the most common metastasis and is a direct contributor to high recurrence and low survival for patients with EOC. Cancer cells exfoliated from primary lesion forms non-adherent MCAs which are the major seeding cells of peritoneal metastases. EOC cells within MCAs exhibit several cancer stem cell-like phenotypes with limited proliferation, yet gain the capacity to reattach to form secondary lesions [7].

Emerging evidence has confirmed that cancer stem cells (CSCs), a small proportion of cancer tissues, are considered as the origin of tumor recurrence and drug resistance [8–10]. CSCs were initially isolated in acute leukemia in 1994 and have been found in various types of cancers since then [11]. CSCs are defined as heterogeneous tumor cell subsets which have pluripotency, self-renewal ability, and efficient tumorigenic capacity [12]. Such subsets can give rise to more differentiated progeny cells with limited proliferative potential which is the reason of chemo-resistance, but also to initiate and “reproduce” such tumors when cells seed other sites to develop metastasis [9]. Thus, CSC-targeted therapy is expected to effectively improve the prognosis of EOC patients [13]. However, studies on CSCs are not thorough enough, and identification and elaboration of CSCs are far from optimal. Therefore, persistent and in-depth exploration of CSC characteristics and mechanisms by which CSCs maintain stemness and progenitor-like properties would facilitate to search for novel effective therapeutic target for ovarian cancer.

Eukaryotic initiation factor 5A2 (EIF5A2) acts as a candidate oncogene and is located on human chromosome 3q26, a region frequently amplified in several tumors [14]. Accumulating evidences show that EIF5A2 plays important roles in enhancing anchorage-independent growth, xenograft tumor growth, increasing cancer cell metastasis, and promoting treatment resistance through multiple pathways [15–18]. In addition, it has been reported that EIF5A2 overexpression enhances the stemness of ESCC cells and contributes to the maintenance of CSC properties via the c-Myc/miR-29b axis in HCC [19, 20]. In ovarian cancer, EIF5A2 elucidated the oncogenic role in the development of ovarian cancer and is considered as an independent predictor of outcome in patients with ovarian carcinoma [21, 22]. However, the role of EIF5A2 in chemo-resistance, stemness, and corresponding regulatory mechanisms in ovarian cancer remains elusive.

In the current study, we performed a series of experiments to investigate the effect of EIF5A2 in regulating the stemness and chemo-resistance of OCSCs and explored the underlying mechanisms by RNA-seq, bioinformatics analysis, and related function experiments. Our results highlight that stemness is enhanced in EOC by EIF5A2/E2F1/KLF4 axis, suggesting a potential innovative CSC-specific therapeutic target, EIF5A2, for the treatment of EOC.

## Methods

### Tissue samples for qRT-PCR and western blot assay

Thirty tumor samples, 20 pairs of primary lesions, and their matched peritoneal MCAs/MCSs from EOC patients were obtained immediately after surgical resection or ascites extraction in the Department of Obstetrics and Gynecology, Qilu Hospital of Shandong University, from February 2019 to March 2020. All patients were pathologically diagnosed with EOC by three pathologists without previous neo-adjuvant chemotherapy, radiotherapy, or immunotherapy. Meanwhile, fimbriae of the fallopian tubes were collected from 18 patients receiving bilateral salpingectomy with benign neoplasms at the same hospital as normal control tissues. This study was examined and approved by the Research Ethics Committee of Shandong University Qilu Hospital (KYLL-2019-2-102). Written informed consents were obtained from all the patients involved in this study before surgery.

### Immunohistochemistry

A total of 76 paraffin-embedded EOC samples were collected from the Department of Pathology, Qilu Hospital, between January 2012 and December 2014. Tissue sections were torrefied for 1.5 h and dewaxed with xylene and ethyl alcohol. This was followed by immunohistochemical staining, according to the protocol instructions. Then, sections were incubated with EIF5A2 antibodies (1:300; ab150439; Abcam; Cambridge, UK) overnight at 4 °C. After incubated with biotin-labeled goat anti-rabbit IgG polymer for 30 min, 3,3′-diaminobenzidine reagent was used to detect positive signals. Antibody positive rate was quantified by Image-Pro Plus software 5.0. The same setting and steps were used for all the analyzed tissue sections to be accurate for the staining reading. Immunoreactive score (IRS) system which ranged from 0 to a maximum score of 12 was used to assess expression of EIF5A2 semiquantitatively (IRS = staining intensity (SI) × percentage of positive cells (PP)). IRS was classified as − (0–3), + (3–6), ++ (6–9), and +++ (9–12). Samples were divided into two groups: low expression (IRS values of 0–6) and high expression (IRS values of 6–12). Staining results and scores were further evaluated by two pathologists.

### Isolation of aggregates/spheroids from ascites fluid

Native aggregates/spheroids from EOC patients’ ascites fluid were isolated by filtration with 40 μm cell strainer (Becton Dickinson), then washed with PBS into collection tubes and resuspended in complete medium to re-differentiation or serum-free medium to culture in vitro. The presence and proportion of epithelial cancer cells was confirmed by flow cytometric detection of EpCAM+/CD45−. The graphs are shown in Additional file 1 (Figure S1).

### Cell culture, sphere-forming, and re-differentiation assay

The human epithelial ovarian cancer cell lines, SKOV3, HO-8910, and HEY, were obtained from the Key Laboratory of Gynecologic Oncology of Shandong Province which were tested and authenticated. The SKOV3 cells and HO-8910 cells were cultured in RPMI-1640 medium. HEY cells were cultured in a DMEM medium. All the medium were added with 10% fetal bovine serum (FBS; BI; Kibbutz Beit-Haemek, Israel), 1% penicillin-streptomycin (Solarbio; Beijing, China). Cells were cultured in a sterile 37 °C incubator with 5% CO_2_. The sphere-forming experiment was carried out in a semi-solid medium. Three thousand adherent cells were resuspended in 1:1 mixed growth factor-reduced Matrigel (BD Biosciences) and serum-free DMEM/F12 (supplemented with B27, EGF, and bFGF). The cell suspension was plated on the edge of the well of the 6-well plate (Corning) and solidified at 37 °C for 20 min, and then 1 ml of serum-free DMEM/F12 (supplemented with B27, EGF, and bFGF) was added. Then renewed the medium every 2 or 3 days for a total of 10 days. The spheroids were cultured in RPMI-1640 medium with 10% FBS after plating in 6-well plate to re-differentiate.

### RNA extraction and quantitative real-time polymerase chain reaction (qRT-PCR)

Total RNA from all clinical specimens and cells were isolated by TRIzol reagent (Invitrogen; CA, USA) according to the manufacturer’s recommendations. After measuring the concentrations of mRNA, total RNA was reverse-transcribed using the reverse transcription cDNA kit (Toyobo; Japan). QRT-PCR was performed using the SYBR Green Real-time PCR Master Mix (Toyobo; Japan) and Light Cycler Roche 480 PCR instrument. The relative mRNA expression was calculated by the 2-ΔΔCt method and normalized to glyceraldehyde 3-phosphate dehydrogenase (GAPDH) expression. The primer sequences are listed in Table S1.

### Western blotting analysis

Total protein from all clinical specimens and cells were extracted using RIPA cell lysis buffer (Beyotime; Shanghai, China) according to the manufacturer’s recommendations. Extracted proteins were separated by sodium dodecyl sulfate-polyacrylamide gel electrophoresis and transferred to polyvinylidene difluoride membranes. The membranes were blocked with 5% fat-free milk dissolved in Tris-buffered saline supplemented with Tween 20 (TBST) for 1 h at room temperature and incubated with primary antibodies against EIF5A2 (1:1000; ab150439; Rabbit; Abcam), E2F1 (1:2000; #3742; Rabbit; Cell Signaling Technology), KLF4 (1:1000; #12173; Rabbit; Cell Signaling Technology), ALDH1A1 (1:1000; #54135; Rabbit; Cell Signaling Technology), and OCT-4 (1:3000; Mouse monoclonal; Proteintech) overnight at 4 °C, GAPDH (1:2000; Rabbit; Multi Sciences) was used as the control. Subsequently, the membranes were incubated with secondary antibody (1:2000; Cell Signaling Technology; Danvers) for 1 h at room temperature. ECL detection system (Amersham Imager 600; Boston) was used for the visualization of protein bands. Semi-quantification of the blots was calculated by Image J software.

### Immunofluorescence staining

EOC peritoneal MCAs and spheroids induced from cell lines were fixed with 4% paraformaldehyde for 30 min at room temperature and subsequently permeabilized with 0.5% Triton X-100 (Solarbio, China) for 10 min. After blocking with 5% BSA in PBS for 30 min at room temperature, cells were incubated with primary antibodies against EIF5A2 (1:200; Rabbit; Abcam) and CD133 (1:400; Mouse; Proteintech) overnight at 4 °C. Cells were stained with Alexa Fluor 594 AffiniPure goat Anti-Mouse IgG (1:50; A8807110; Yeasen) and Alexa Fluor 488 AffiniPure goat Anti-Rabbit IgG (1:100; A9825370; Yeasen) for 1 h at room temperature in the dark after thorough washing with PBS. Nuclei were stained with 4′,6-diamidino-2-phenylindole (DAPI; Boster Biological Technology Co Ltd.; Wuhan, China) for 5 min. Immunofluorescence images were observed and collected with a fluorescence microscope (DP72; Olympus; Tokyo, Japan).

### SiRNA, plasmid extraction, and lentivirus transfection

Si-EIF5A2, si-E2F1, and si-KLF4, along with RNAi negative controls, were purchased from Genepharma (Shanghai, China). SiRNA silencing was realized by transient transfection with INTERFERin (Polyplus, Shanghai, China) according to the manufacturer’s instructions. The siRNA sequences are detailed in Table S1. The plasmids EIF5A2, E2F1, and KLF4 were purchased from Genechem (Shanghai, China). After transfecting 1 μg plasmid into competent cells, shake flask culture with 100 μg/ml ampicillin to amplify bacteria. Then plasmids were extracted with plasmid extraction kit (Omega, USA) and the purity and concentration were detected with spectrophotometer (Thermo Fisher Scientific Inc., USA). Lentivirus expressing KLF4 packaged with psPAX2 (Addgene, USA) and pMD2G (Addgene, USA) were produced in HEK293T cells by use of Lipofectamine 2000 transfection agent (Invitrogen, USA). Lentiviruses carrying short hairpin RNA (shRNA) targeting EIF5A2 lentiviral vectors were designed and synthesized by GeneChem. The RNAi and NC sequences are listed in Table S1. Twenty-four hours after infection with lentivirus, stably knocked down (KD) and overexpressing (OE) cells were selected in a medium containing 2μg/mL puromycin for a week. The efficiency of knockdown or overexpression was detected by qRT-PCR and western blotting.

### Cell viability assays

After transfected with siRNA or plasmid for 48 h, cells were cultured in SFM soft agar condition for 7 days. Then 8000 cells/well digested from spheroids were seeded in 96-well plates (Corning), followed by exposure to cisplatin at various concentrations for 36 h. Then 10 μL CCK8 (Dojindo Laboratories, Kumamoto, Japan) was added to each well, and cells were incubated at 37 °C for another 2 h. For cell survival and proliferation assay, 2000 cells/well were seeded in 96-well plates. One, three, or five days after incubation, cell viability was measured using CCK8. Varioskan Flash microplate reader (Thermo Scientific) was used to measure optical density (OD) values at 450 nm wavelength. Drug sensitivity was determined from cell viability calculated by absorbance values compared with the control group.

### Flow cytometry analysis

Spheroids induced from SKOV3 and HO-8910 cells were filtered through 40 μm filter to remove cell fragments, centrifuged and digested with trypsin. 1 × 10^6^ cells were resuspended in 100 μL PBS contained 0.1% BSA after washed. Then the cells were stained with anti-CD44-APC**/**PE (eBiosciences, Austria), anti-ALDH-FITC (eBiosciences)**,** anti-CD24-PI (eBiosciences) antibodies, anti-CD133-PE or IgM isotype control antibodies (eBiosciences), and incubated under dark condition for 20 min at room temperature according to the manufacturer’s protocol. Cells exhibiting ALDH+/CD44+ and ALDH−/CD44- phenotype were isolated using FACSAria II flow cytometer. Ratio analysis was performed by Flow Jo 7.6 software.

### RNA-sequencing analysis

Total RNAs were extracted from SKOV3 cells transfected with si-EIF5A2 or negative control. The RNA amount and purity of each sample were quantified using NanoDrop ND-1000 (NanoDrop, Wilmington, DE, USA). The RNA integrity was assessed by Bioanalyzer 2100 (Agilent, CA, USA) with RIN number > 7.0, and confirmed by electrophoresis with denaturing agarose gel. 2 × 150 bp paired-end sequencing (PE150) of each sample was performed on an Illumina Novaseq™ 6000 (LC-Bio Technology CO., Ltd., Hangzhou, China) following the vendor’s recommended protocol for mRNA library construction and sequencing. After the final transcriptome was generated, StringTie and ballgown were used to estimate the expression levels of all transcripts and perform expression level for mRNAs by calculating FPKM (FPKM = [total_exon_fragments/mapped_reads(millions) × exon_length(kB)]). The differentially expressed mRNAs were selected with fold change > 2 or fold change < 0.5 and *p* value < 0.05 by R package edgeR or DESeq2, and then analysis GO enrichment and KEGG enrichment to the differentially expressed mRNAs.

### Luciferase activity assay

To investigate E2F1 binding sites in the KLF4 promoter, three predicted wild-type binding sites and a mutant binding site of E2F1 were separately cloned into pGL3 vector (Promega) to perform luciferase activity assay. Renilla luciferase pRL-TK reporter vector (Promega) was used to normalize luciferase activity. Cells were seed in 24 well plates and transfected with E2F1 plasmids or control vector. After 48 h, cells were collected into 96 well plates for luciferase assays. The ratio of Firefly luminescence to the Renilla luminescence is the relative luciferase activity.

### In vivo xenograft experiments

The animal experiment was conducted according to national guidelines for the use of laboratory animals and was approved by the Laboratory Animal Ethical and Welfare Committee of Shandong University Cheeloo College of Medicine (Approval number: 19074). BALB/c mice (female, 4-week-old) were randomly assigned to each treatment group. For different gradient xenograft tumorigenicity experiments, 1 × 10^5^, 1 × 10^4^, or 5 × 10^3^ SKOV3 spheroid cells transfected with shEIF5A2 or shNC were resuspended in 100 μl PBS and injected into the right flank of mice, which were then monitored weekly for 5 weeks. For in vivo drug sensitivity experiment, 1 × 10^6^ SKOV3 cells transfected with shEIF5A2 or shNC were injected into the right flank of mice. Cisplatin treatment was performed via intraperitoneal injection of 5 mg/kg cisplatin in 0.9% NaCl at days 7, 14, 21, and 28. For in vivo rescue experiment, 1 × 10^6^ SKOV3 cells transfected with NC or shEIF5A2 or shEIF5A2 + KLF4-plasmid were injected into the right flank of mice. Tumor volume was calculated by the formula: V (tumor) = 0.5 × *D* × (*d*^2^) (*D* = largest length, *d* = smallest width).

### Statistical analysis

Statistical comparison between groups was performed using two-tailed Student’s *t* tests. The correlation between EIF5A2 expression with clinicopathological features and possible downstream molecules was analyzed with the two-tailed Spearman correlation analysis. The EIF5A2 survival analysis was conducted by the Kaplan-Meier method with log-rank test. Differences were considered statistically significant when *P* < 0.05 (* *P* < 0.05, ** *P* < 0.01, and *** *P* < 0.001). All experiments were repeated three times and data were shown as mean ± standard error of mean (SEM). All the statistical analysis was performed using SPSS Statistics 24.0 (Armonk, NY) and GraphPad Prism 7.0 (USA).

## Results

### EIF5A2 is upregulated in EOC specimens and its expression is associated with chemo-resistance and poor prognosis

**Fig. 1:**
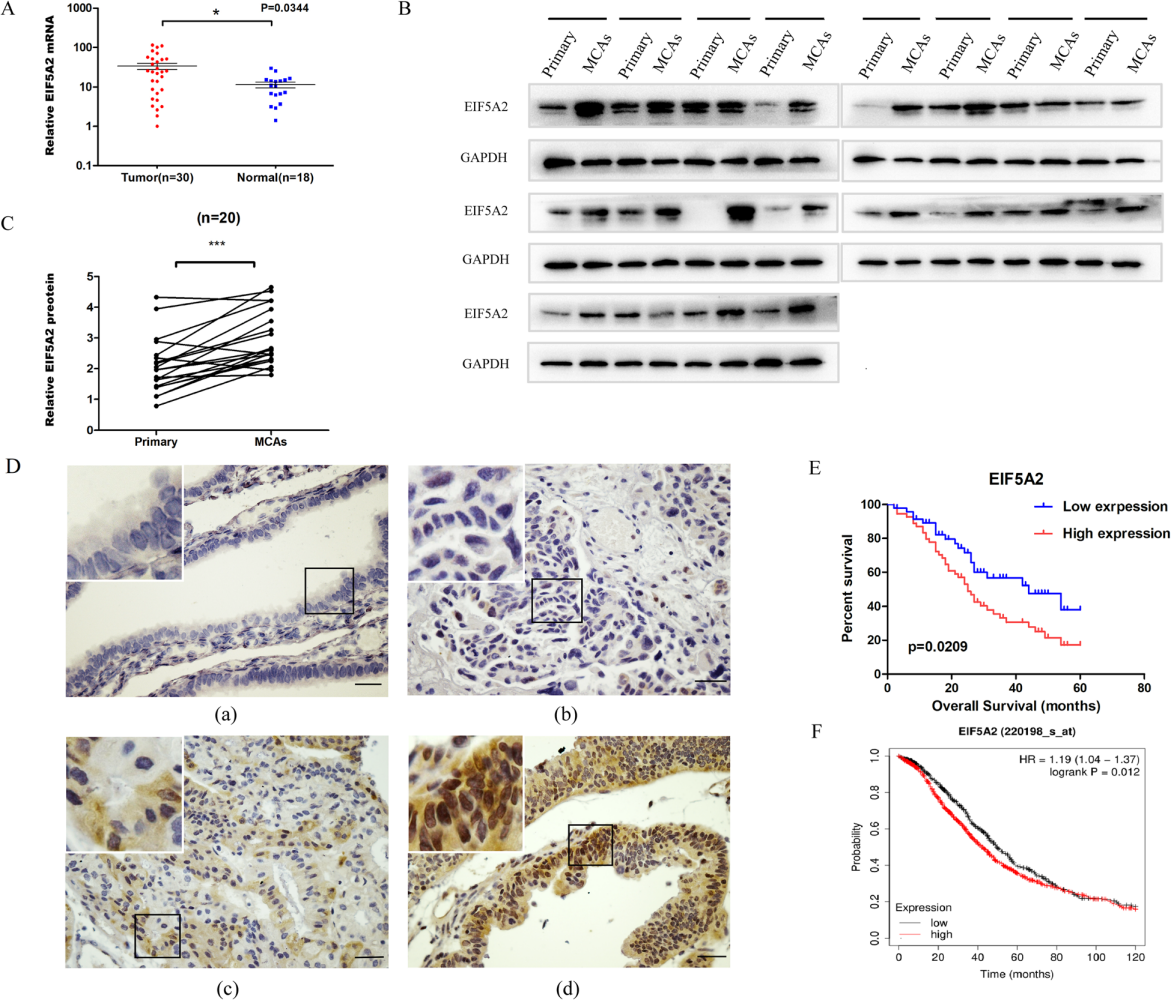
EIF5A2 is upregulated in EOC specimens and its expression is associated with chemo-resistance and poor prognosis. **A** qRT-PCR was used to detect the expression of EIF5A2 in 30 EOC tissues and 18 normal control tissues. **B** Western-blot assay bands of 20 pairs primary tissues and MCAs in ascites of EOC patients using Image J. **C** Quantitation of protein expression. Statistical analysis was performed using Student’s *t* test. **D** Immunohistochemical staining was used to detect the expression of EIF5A2 in 76 EOC tissue samples. Scale bar = 50 μm. (a) Normal expression of EIF5A2 was observed in a normal epithelium of fallopian tube tissue. (b) Low-expression of EIF5A2 was detected in an ovarian carcinoma (case 24). (c) Overexpression of EIF5A2 was detected in an ovarian carcinoma (case 28), in which about 60% of tumor cells showed moderate positive staining of EIF5A2. (d) Another ovarian carcinoma (case 45) showed overexpression of EIF5A2, in which 90% of tumor cells had strong positive staining of EIF5A2. **E** Kaplan-Meier analysis of EOC patients with low- or high-EIF5A2 expression. **F** The association of EIF5A2 expression with EOC patients’ survival in 1657 advanced EOC patients from GEO and TCGA database. All the data represent the means ± SD; *p < 0.05, **p < 0.01, and ***p < 0.001

To detect the expression of EIF5A2 in EOC development, we analyzed EIF5A2 mRNA level in 30 EOC patients and 18 normal control tissues by qRT-PCR. The expression of EIF5A2 was significantly upregulated in EOC tissues compared to fallopian tube tissues (*P* = 0.0344)**(**Fig. 1A). Furthermore, we detected EIF5A2 expression in 20 pairs of primary lesions and MCAs in ascites of EOC patients. The results showed that protein level of EIF5A2 in MCAs were higher than that in primary lesions (*P* < 0.001) (Fig. 1B, C). To explore the alteration of EIF5A2 in EOC progression, the EIF5A2 expression was examined in 76 paraffin-embedded EOC tissues by immunohistochemistry analysis. The clinicopathological parameters of EOC patients were summarized in Table 1. The EIF5A2 expression scores ranged from 0 to 11, with a median of 6 (Fig. 1D). The high EIF5A2 expression was significantly associated with clinical response (*P* = 0.039) and platinum sensitvity (*p* = 0.014). No statistical significance was found in the correlation between its expression and other parameters including age, histological type, histological grade, lymphatic metastasis, FIGO stage, and residual disease. Kaplan-Meier survival analysis indicated that the patients with a high EIF5A2 expression had a significantly shorter survival (OS, *n* = 76, *P* = 0.0209, Fig. 1E) than those with a low EIF5A2 expression, which was consistent with the survival data analyzed in GEO and TCGA database (OS, *n* = 1657, *P* = 0.012, Fig. 1F).

**Table 1:** Correlations between EIF5A2 expression and clinicopathologic characteristics of EOC patients

Characteristics	All cases	EIF5A2 protein		***P*** value
		Low expression	High expression	
Age (years, mean)	76	57	55	0.28
Histological type				0.201
Serous	61	25 (41.0%)	36 (59.0%)	
Others	15	9 (60.0%)	6 (40.0%)	
Histological grade				0.724
Well/moderate	10	5 (50.0%)	5 (50.0%)	
Poorly	66	29 (43.9%)	37 (56.1%)	
Lymphatic metastasis				0.108
No	63	30 (47.6%)	33 (52.4%)	
Yes	13	4 (30.8%)	9 (69.2%)	
FIGO stage				0.263
IIIc	58	28 (48.3%)	30 (51.7%)	
IV	18	6 (33.3%)	12 (66.7%)	
Residual disease				0.381
≤ 10 mm	59	28 (47.5%)	31 (52.5%)	
> 10 mm	17	6 (35.3%)	11 (64.7%)	
Clinical response				0.039
Complete	68	33 (48.5%)	35 (51.5%)	
Partial	7	1 (14.3%)	6 (85.7%)	
Non-response	1	0 (0.0%)	1 (100.0%)	
Platinum sensitivity				0.014
Sensitive	60	31 (51.7%)	29 (48.3%)	
Resistant*	16	3 (18.8%)	13 (81.2%)	

*Platinum-resistant was defined as those with relapse within 6 months after primary chemotherapy or progress during the primary chemotherapy

### Upregulated EIF5A2 expression in OCSC-like cells

**Fig. 2:**
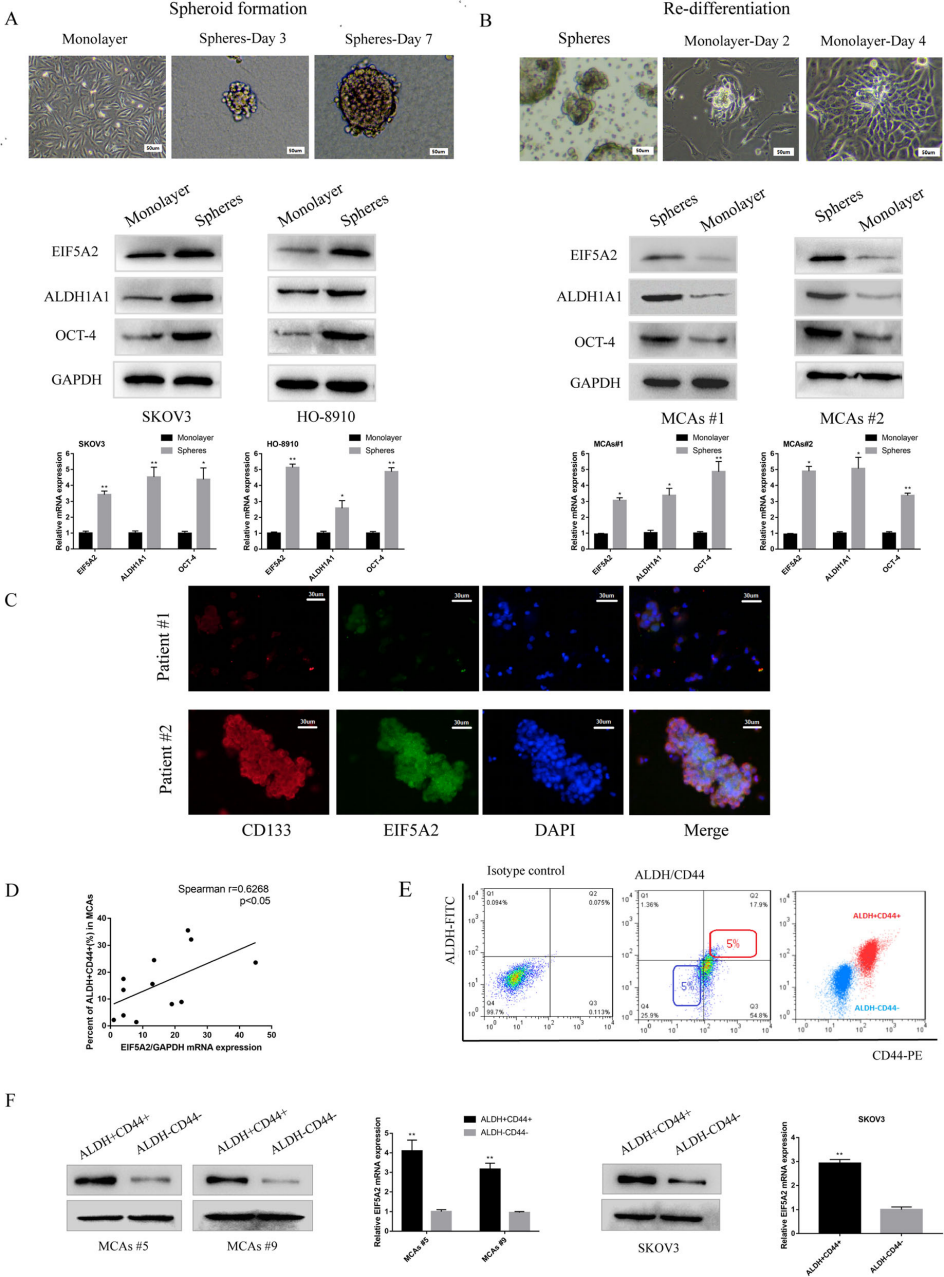
Upregulated EIF5A2 expression in OCSC-like cells. **a** Protein expression of EIF5A2, OCT-4, and ALDH1A1 of parental SKOV3, HO-8910 cells (monolayer), day 7 spheroid cells derived from SKOV3, HO-8910 cells growing in serum-free medium with growth factors (spheres), measured by western-blot. **b** Protein expression of EIF5A2, OCT-4, and ALDH1A1 of MCAs from two EOC ascites (spheres), adherent cells after re-differentiation growing in serum-complete medium (monolayer). **c** Immunofluorescent staining of CD133 (red), EIF5A2 (green), and their co-localization (yellow) in EOC malignant cell from ascites. **d** Correlation analysis demonstrating that overexpression of EIF5A2 was positively correlated with percent of ALDH+CD44+ subpopulation cells in MCAs from EOC patients. (*r* = 0.6268, *p* < 0.05). **e** Isolation of ALDH+CD44+ and ALDH−CD44− subpopulaiton cells by FACS. (Left) Isotype control. (Middle) The top 5% cells showing the highest staining for ALDH+CD44+ or bottom 5% with minimal staining for ALDH-CD44- cells were collected. (Right) Purity of ALDH and CD44 was confirmed via post sorting using flow cytometry. **f** Relative EIF5A2 mRNA and protein expression of ALDH+CD44+ and ALDH-CD44- subpopulation cells in MCAs #5, MCAs #9, and SKOV3 cells via qPCR and immunoblotting, respectively. All the data represent the means ± SD; *p < 0.05, **p < 0.01, and ***p < 0.001

Since EIF5A2 was upregulated in EOC peritoneal MCAs, associated with chemo-resistance, and served as a CSC modulator in other tumors, we explored whether EIF5A2 played a role in maintaining CSC properties in EOC. In order to figure it out, we detected EIF5A2 expression in 3 EOC cell lines. According to the results **(**Figure S3 a,b), we chose SKOV3 cell lines to create the EIF5A2 knockdown model and HO-8910 cell lines to create the EIF5A2 overexpression model. Then, we cultured the two cell lines in the serum-free medium to induce cells into spheroid clusters and observed the alteration of EIF5A2 protein expression in the process of spheroid formation. As shown in Fig. 2a, we found that the expression of EIF5A2 protein presented an increasing trend when adherent cells gradually became suspensive, denser, and multi-cellular spheroids. Consistently, the expression levels of ALDH1A1 and OCT-4, two stem-related markers, were increased during this process. In contrast, we also observed the change of EIF5A2 protein expression when MCAs of EOC ascites were re-differentiated in medium with 10% FBS. As shown in Fig. 2b, the expression of EIF5A2 protein presents a decreasing trend following EOC cell growth from spheroid into the adherent state. Consistently, during this process, the expression of ALDH1A1 and OCT-4 were decreased. Then, co-localization of EIF5A2 with CSC marker, CD133, in EOC ascites specimens were confirmed by immunofluorescence (Fig. 2c). Previous study reported that ascites-derived tumor cells exhibit CSC properties and ALDH combined with CD44 were used as markers to characterize OCSC-like cells [23]. To delineate the ALDH+CD44+ cells in EOC, we measured the percentage of ALDH+CD44+ cells in MCAs samples from EOC ascites and 3 ovarian cancer cell lines (Figure S2, Table S2). Correlation analysis showed that overexpression of EIF5A2 was positively correlated with percentage of ALDH+CD44+ cells (Fig. 2d). ALDH+CD44+ cells from 2 samples (patient #5, 9) and SKOV3 cells sorted by flow cytometry were used for further study (Fig. 2e). The data showed that the level of EIF5A2 expression was significantly higher in ALDH+CD44+ cells than those of ALDH−CD44− cells (Fig. 2f). Our results indicated that EIF5A2 expression is upregulated in OCSC-like cells.

### EIF5A2 positively modulates stem-like properties in ovarian cancer cells

**Fig. 3:**
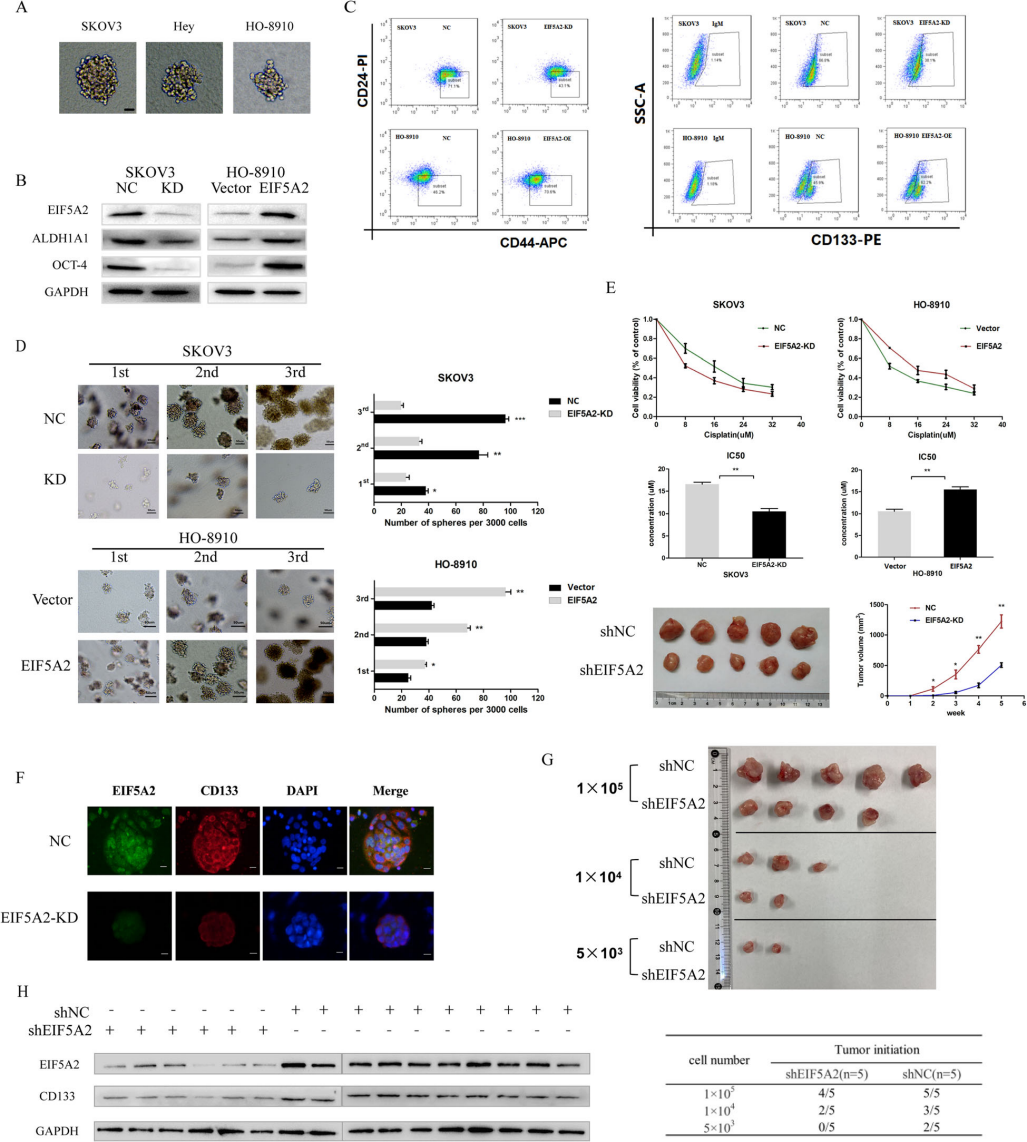
EIF5A2 positively modulates stem-like properties in ovarian cancer cells. SKOV3 and HO-8910 cells were transfected with shEIF5A2 or EIF5A2 plasmid, and cultured in serum-free culture condition for 7 days. **a** Morphology of spheroids derived from SKOV3, Hey, and HO-8910 cells are shown (bar = 40um). **b** The expression of EIF5A2 and stem-related markers, OCT-4 and ALDH1A1, were detected by Western blotting analysis. **c** The proportion of CD44+CD24− and CD133+ phenotype in SKOV3 spheroids and HO-8910 spheroids was analyzed by FCM (Figure S5 Quantitative analysis of Flow Cytometry). **d** Single-cell suspensions with 3000 cells were seeded in 6-well culture plates and cultured in semi-solid serum-free medium for 5 days. The number and size of spheroids formed was determined via microscopy, and representative pictures are shown. Representative images and the numbers of NC, EIF5A2-KD SKOV3 and vector, EIF5A2-OE HO-8910 cell-derived spheroids from three serial passages were compared (Figure S6 Quantitative analysis of spheroid formation ability). **e** NC, EIF5A2-KD SKOV3 and vector, EIF5A2-OE HO-8910 cells were treated with cis-platinum for 36 h. The cell viability was measured through a CCK-8 assay, and the data are presented as the fold change relative to the treatment-free groups (left: cell viability curve, right: IC50 quantitative analysis). The effect of EIF5A2 knockdown on the drug sensitivity of ovarian cancer cells in vivo was analyzed by inoculating SKOV3 cells into nude mice. Data represents the mean ± standard error of three independent experiments. **f** Immunofluorescent staining of CD133 (red), EIF5A2 (green) and their co-localization (yellow) in NC and EIF5A2-KD SKOV3-spheroids. **g** SKOV3 cells were transfected with shNC or shEIF5A2 for 48 h, cultured in spheroid culture conditions for 7 days, and then injected into BALB/c mice (female, 4-week-old) (*n* = 5 in each group). All mice were sacrificed at week 5 and the tumor incidence was evaluated. Subcutaneous tumors are shown. **h** Western blot assay were used to detect the protein levels of EIF5A2 and CD133 in the formed tumors. All the data represent the means ± SD; *p < 0.05, **p < 0.01, and ***p < 0.001

Since EIF5A2 expression was detected to be upregulated in EOC peritoneal MCAs, spheroid cells from cell lines, and ALDH+CD44+ subpopulaiton. EIF5A2 may act as a potential modulator in maintaining CSC-like property of ovarian cancer cells. The spheroid formation capacity of three cell lines was conducted first (Fig. 3a) (Figure S3,c). Next, we transfected specific plasmid or siRNA to overexpress EIF5A2 in HO-8910 cells or suppress EIF5A2 expression in SKOV3 cells, which were cultured in a serum-free medium with growth factors to form spheroids for 7 days. As Fig. 3b and Figure S4 showed, EIF5A2 mRNA and protein levels were successfully overexpressed by specific plasmid and inhibited by siRNA. As we expected, EIF5A2 knockdown inhibited the expression of stem-like markers, ALDH1A1 and OCT4, in spheroids (Fig. 3b), decreased the proportion of CD44+/CD24− cells from 71.1 to 43.1% and CD133+ cells from 66.8 to 38.1% in SKOV3 spheroids (Fig. 3c) (Figure S5), weakened the spheroid forming ability of cells (Fig. 3d) (Figure S6), increased the sensitivity of cells to cisplatin in vitro and in vivo (Fig. 3e). In contrast, EIF5A2 overexpression upregulated the expression of stem-related markers, ALDH1A1 and OCT4, in spheroids (Fig. 3b), and increased the proportion of CD44+/CD24− cells from 46.2 to 70.6% and CD133+ cells from 45.9 to 62.2% in HO-8910 spheroids (Fig. 3c) (Figure S5), strengthened the spheroid forming ability of cells (Fig. 3d)(Figure S6), and decreased the sensitivity of cells to cisplatin (Fig. 3e). Since cell survival and proliferation could affect the effects of EIF5A2 on spheroid assay, we further evaluated the cell viability after EIF5A2 knockdown and overexpression. EIF5A2 knockdown in SKOV3 and EIF5A2 overexpression in HO-8910 did not show alteration in cell viability 1 day after incubation. We also found the cell viability of EIF5A2-depleted SKOV3 as 0.81–0.85-fold and EIF5A2-upregulated HO-8910 as 1.09–1.24-fold after 5 days incubation compared to control as determined using CCK8 (Figure S7), suggesting that the decreased or enhanced sphere-forming capacity would not result primarily from cell death or cell proliferation. Furthermore, co-localization analysis by immunofluorescence confirmed that the silence of EIF5A2 observably reduced the expression of CD133 in sphere cells (Fig. 3f). Efficient tumorigenesis in vivo is one of the most important features of cancer stem cells [24]. We performed xenograft tumorigenicity assays in nude mice using different gradients of CSC subpopulations enriched from SKOV3-shNC or shEIF5A2 spheroid cells. The assays showed that the volume and number of the xenograft tumors formed by SKOV3-shNC spheroid cells were always higher than those formed by shEIF5A2 spheroid cells (Fig. 3g). These data showed that EIF5A2 knockdown weakened the CSC subpopulation-induced tumorigenicity. We also measured the level of CD133 protein expression in the control (6 formed tumors) and EIF5A2-KD (10 formed tumors) group (Fig. 3h) and found that overexpression of EIF5A2 was correlated with CD133 expression (Figure S8). Together, these results suggest that EIF5A2 positively modulates stem-like characteristics in ovarian cancer cells.

### KLF4 participates in the EIF5A2-mediated modulation of CSC properties in EOC

**Fig. 4:**
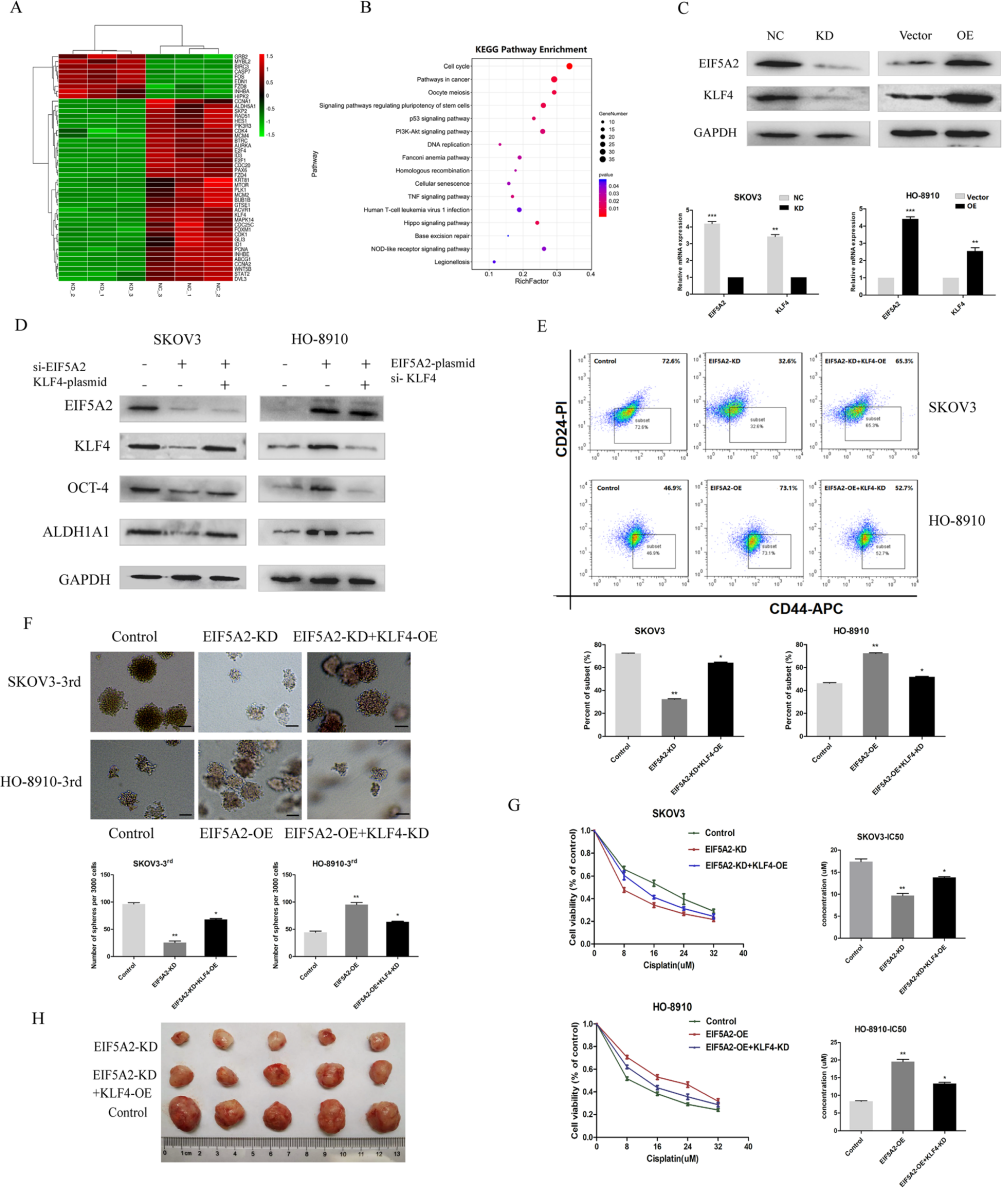
KLF4 participates in the EIF5A2-mediated modulation of CSC properties in EOC. **a** RNA-sequencing data from the analysis between NC and si-EIF5A2 SKOV3 cells. The cluster map generated by DESeq2 software analysis displayed a series of significant differentially expressed, top pathways enriched genes (fold change > 1.5) genes (Padj< 0.05). Downregulated genes are shown as green color and upregulated gene are shown as red color. **b** KEGG analysis of enriched pathways on the basis of transcriptome sequencing. **c** qRT-PCR and immunoblotting analysis of the expression of KLF4 in shNC, shEIF5A2 SKOV3 cells and vector, EIF5A2-plasmids HO-8910 cells. **d** SKOV3 cells were transfected with NC, si-EIF5A2 or si-EIF5A2 plus KLF4 overexpression plasmid, HO-8910 cells were transfected with a control vector, EIF5A2 overexpression plasmid, or EIF5A2 overexpression plasmid plus si-KLF4, and cultured in spheroid culture conditions for 7 days. The indicated stem-markers, OCT-4 and ALDH1A1, of SKOV3 and HO-8910 spheroids were detected by western blotting. **e** The proportion of CD44+/CD24− phenotype in SKOV3 and HO-8910 spheroids were detected by FCM (up: representative images, down: quantitative analysis). **f** Sphere-formation (sphere > 50 μm) was assessed (scale bars, 50 μm). **g** The sensitivity of cells to cisplatin was analyzed by CCK8, and the data are presented as the fold change relative to the treatment-free groups (left: cell viability curve, right: IC50 quantitative analysis). **h** The effect of EIF5A2 knockdown and EIF5A2 knockdown plus KLF4 overexpression on the growth of ovarian cancer cells in vivo was analyzed by inoculating SKOV3 cells into nude mice. All the data represent the means ± SD; *p < 0.05, **p < 0.01, and ***p < 0.001

To explore the specific mechanism of EIF5A2 in EOC, transcriptome sequencing was performed (GSE162198), and several significantly altered and top pathways enriched genes (fold change > 1.5) were selected (Fig. 4a). Surprisingly, we noticed that pathways regulating pluripotency of stem cells were one of the most enriched pathways performed by KEGG analysis in our RNA seq data (Fig. 4b). Of those, KLF4 was shown to be particularly downregulated (log2(fc) = − 3.37; *p* = 0.00) and has been demonstrated to be associated with tumorigenesis and cancer stemness. Thus, we speculated that EIF5A2 might promote the tumor initiation and cancer stemness of EOC cells by activating KLF4 expression. To validate it, we first knocked down and overexpressed EIF5A2 in EOC cell lines and performed western blotting and qRT-PCR to examine KLF4 expression. EIF5A2 depletion decreased KLF4 expression at both the mRNA and protein levels (Fig. 4c). Consistent with these results, the overexpression of EIF5A2 in HO-8910 cells increased KLF4 mRNA and protein expression (Fig. 4c).

To investigate whether KLF4 is required for EIF5A2-mediated modulation of CSC properties in EOC, rescue experiments were conducted. We overexpressed KLF4 after EIF5A2 knockdown in SKOV3 cells silenced KLF4 expression after EIF5A2 overexpression in HO-8910 cells and found that KLF4 overexpression or knockdown reversed, at least partially, stem-like properties that were modulated by EIF5A2. As Fig. 4d showed, inhibited OCT-4 and ALDH1A1 expression were partially reversed in SKOV3 cells and upregulated OCT-4 and ALDH1A1 expression were partially inhibited in HO-8910 cells. The proportion of CD44+/CD24− cells in SKOV3 spheroids varied from 72.6 to 32.6% following EIF5A2 knockdown, then rose to 65.3% by KLF4 overexpression. In contrast, the proportion of CD44+/CD24− cells in HO-8910 spheroids varied from 46.9 to 73.1% following EIF5A2 overexpression, then decline to 52.7% by KLF4 knockdown (Fig. 4e). In addition, weakened spheroid formation ability of SKOV3 cells caused by EIF5A2 knockdown was enhanced again by KLF4 overexpression, while KLF4 downregulation inhibited spheroid formation ability of HO-8910 cells strengthened by EIF5A2 overexpression (Fig. 4f). Moreover, EIF5A2 knockdown increased the sensitivity of SKOV3 cells to chemotherapeutic drugs, but KLF4 overexpression partially attenuated it. In contrast, after siKLF4 treatment, EIF5A2-induced chemo-resistance was inhibited (Fig. 4g). Finally, in vivo xenograft experiment showed that when KLF4 was introduced into EIF5A2-KD SKOV3 cells, the tumorigenicity was substantially re-enhanced (Fig. 4h).

### EIF5A2 modulates the expression of KLF4 through the transcription factor E2F1

**Fig. 5:**
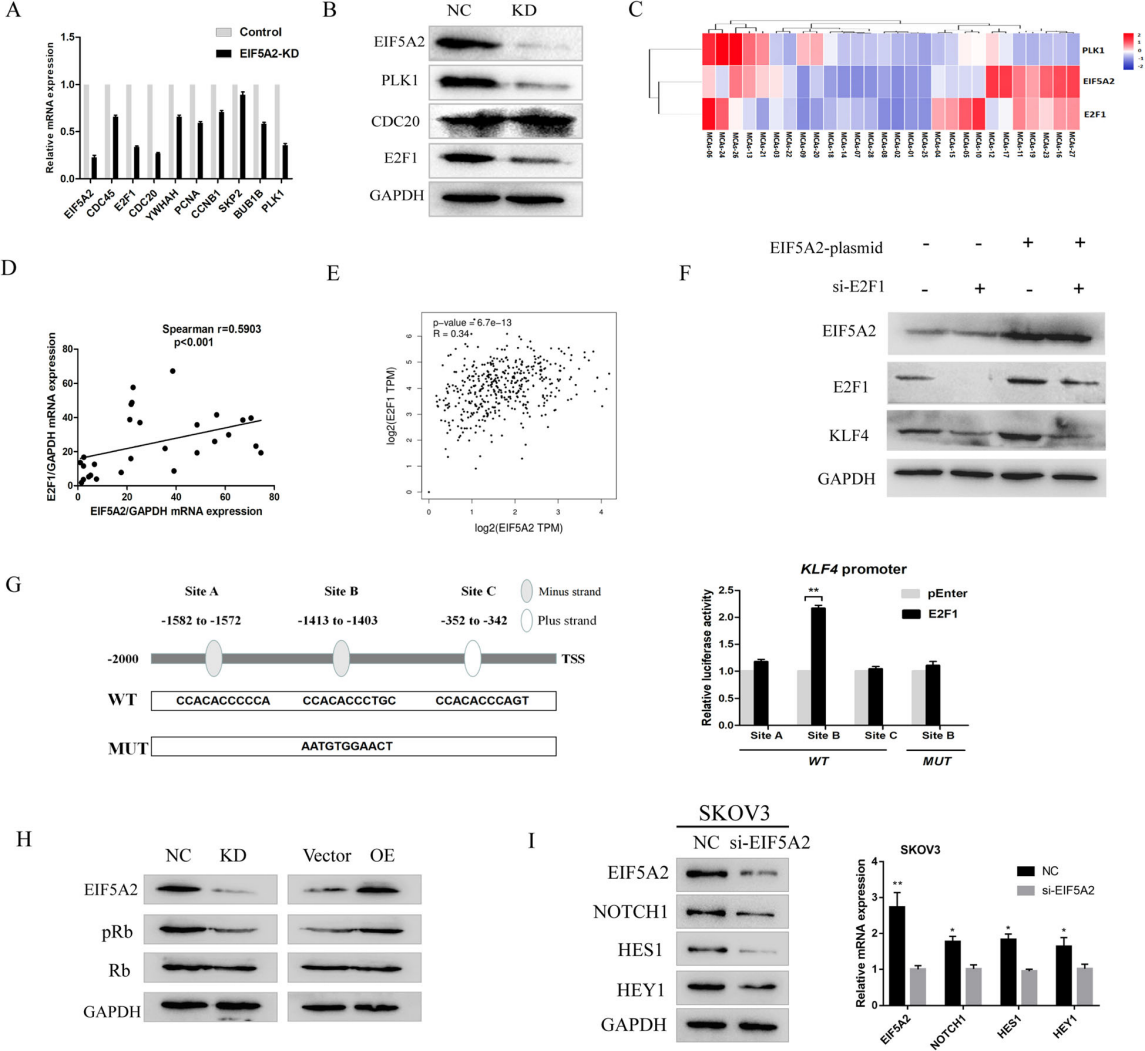
EIF5A2 modulates the expression of KLF4 through the transcription factor E2F1. **a** The mRNA levels of 9 candidate genes in SKOV3 cells with EIF5A2 knockdown were detected by qRT-PCR. **b** Knockdown of EIF5A2 substantially downregulated PLK1 and E2F1 expression in SKOV3 cells detected by western blotting. **c** Heatmap of EIF5A2, PLK1, and E2F1 mRNA expression of 28 MCAs samples detected by qRT-PCR. **d** The association between relative EIF5A2 and E2F1 mRNA levels in 28 MCAs samples was analyzed according to the Spearman-correlation factor. **e** The association between relative EIF5A2 and E2F1 mRNA levels from TCGA database. **f** Expression of KLF4 in SKOV3 cells transfected with empty vector, EIF5A2 plasmid, si-E2F1, or EIF5A2 plasmid plus si-E2F1. **g** The WT box indicates that there are 3 potential E2F1 binding sites within 2000 bp upstream of the KLF4 promoter region predicted by JASPAR, and the MUT box indicates the corresponding mutation sequence. The double-luciferase reporter gene experiment confirmed that only Site B transcriptional activity was significantly increased among the 3 binding sites, and the difference in luciferase activity disappeared after mutation. **h** Expression of pRb, Rb in SKOV3 cells after EIF5A2 downregulation and in HO-8910 cells after EIF5A2 upregulation. **i** Knockdown of EIF5A2 substantially downregulated Notch1, Hes1, and Hey1 expression in SKOV3 cells detected by western blotting and qRT-PCR

To further explore the regulatory mechanism of EIF5A2 in KLF4 transcription, we analyzed the RNA seq data to investigate whether some transcriptional events were involved in the differentially expressed genes following EIF5A2 depletion. As Fig. 4b showed, cell cycle progression was among the top enriched pathways. Then, 10 candidate genes involved in the pathway which possess binding sites to the KLF4 promoter region predicted in the JASPAR database were selected for further verification by qRT-PCR (Fig. 5a). The results indicated E2F1, CDC20, and PLK1 as possible targets of EIF5A2 (fold change > 2.0). Subsequently, western blotting assay further validated that the expression of E2F1 and PLK1 but not CDC20 were regulated by EIF5A2 (Fig. 5b). In addition, a significant positive correlation between the protein level expression of EIF5A2 and E2F1 was evaluated in 28 EOC peritoneal MCAs cases (Fig. 5c, d), which is consistent with correlation analysis from TCGA database (Fig. 5e). There was no significant positive correlation between PLK1 and EIF5A2 expression (*r* = 0.1618, *p* = 0.4109) (Fig. 5c). In order to explore whether EIF5A2 regulates KLF4 in an E2F1-dependent manner, western blot assay was conducted. As data showed, after siE2F1 treatment, the protein level of KLF4 was downregulated. Furthermore, E2F1-KD inhibited the overexpression of KLF4 in EIF5A2-OE cells (Fig. 5f). Next, we noticed that E2F1 could bind to several sequences in the KLF4 promoter region (2 kb upstream of the transcriptional start site) according to the JASPAR database (relative profile score threshold = 90%) (Fig. 5g). Therefore, we hypothesized KLF4 as a direct transcriptional target of E2F1. Subsequently, we constructed wild-type and mutant reporter gene vectors containing E2F1 binding sites in the KLF4 promoter region and tested the binding activities of these potential binding sites with the aid of dual luciferase reporter gene experiments. The results showed that there is a significant increase in luciferase activity at one site (CCACACCCTGC), and after mutation of this site (AATGTGGAACT), the difference disappears. This suggests that E2F1 positively regulates the expression of KLF4, which is consistent with the above experimental results. The CDKs/pRb pathway plays an important role in regulating the transcriptional activity of E2F1, in which pRb loses its suppression on E2F1 and activates E2F1 targets [25, 26]. In our RNA seq data, CDK1 and CDK4 were downregulated after EIF5A2 knockdown. To address whether EIF5A2 affects E2F1 also through inactivation of pRB, we detected the phosphorylation of Rb upon EIF5A2 depletion or overexpression. Western blotting indicated that EIF5A2 depletion decreased Rb phosphorylation levels, while EIF5A2 overexpression increased Rb phosphorylation levels (Fig. 5h). These results indicate that EIF5A2 might regulate E2F1, at least in part, through CDKs/pRB pathway, and then transcriptionally activate KLF4 expression.

Given that KLF4 is only able to partially reverse the KD or OE effect of EIF5A2, other CSC-related molecules involved in differential expressed RNA seq data were verified by qRT-PCR (Figure S9). We found that HES1, a primary target gene of the Notch pathway, was downregulated (fold change > 2.0) other than KLF4. Next, we examined the effects of EIF5A2 induction on the activation of the Notch pathway. As the data (Fig. 5i) showed, the downregulation of EIF5A2 decreased the level of Notch1, Hes1, and Hey1 in SKOV3 cells. Consistent with these results, overexpression of EIF5A2 activated Notch pathway in HO-8910 (Figure S10).

## Discussion

Multiple types of human cancers present aberrant expression of EIF5A2, which is critical for tumor growth, metastasis, and treatment resistance. In this study, we uncover the upregulation of EIF5A2 in peritoneal multicellular spheroids/aggregates compared with primary lesions, disclose the correlation with chemo-resistance, and poor prognosis in EOC patients, and we provide evidence that EIF5A2 maintains CSC properties through E2F1/KLF4 pathway, suggesting that EIF5A2 might be a potential CSC-specific therapeutic target.

Previous studies have illustrated that EIF5A2 is highly expressed in ovarian tumor tissues and acts as an oncogenic role in EOC patients [21, 22], but the effect of EIF5A2 in CSCs and specific mechanism in EOC remains largely unclear. Therefore, we detected the expression of EIF5A2 in the process of spheroid-formation of SKOV3 and HO-8910 cells and re-differentiation of peritoneal MCAs cells which are often characterized by the expression of stemness-associated markers and genes [27] and found that the expression of EIF5A2 and stem markers (OCT-4, ALDH1A1) showed consistently altered tendency during two opposite processes. Further, we downregulated and overregulated the expression of EIF5A2 in ovarian cancer cells and found that the expression of the related stem genes, spheroid formation ability, chemotherapeutic drug sensitivity, and in vivo different gradients tumorigenicity were attenuated or strengthened, suggesting that EIF5A2 functions in maintaining the stemness of ovarian cancer cells, which is demonstrated for the first time.

To further explore downstream molecular pathways involved in stemness regulation of EIF5A2, we used RNA-sequencing assay, KEGG analysis, qRT-PCR, western blot to examine differentially expressed genes, and found that the KLF4 expression was decreased after EIF5A2 downregulation in EOC cells. As one of the four factors (SOX2, C-Myc, OCT-4, and KLF4) required for reprogramming adult fibroblasts and skin melanocytes into induced pluripotent stem cells (iPS), KLF4 plays an essential role in tumor initiation and cancer stemness [28, 29]. Our function rescue experiments showed that some deprived CSC properties induced by EIF5A2 knockdown, such as decreased expression of stem markers, inhibited spheroid forming ability, and reduced chemo-resistance and tumorigenicity, were partially reversed by KLF4 overexpression, suggesting that KLF4 participates in the process of EIF5A2 modulating stemness in ovarian cancer cells. In subsequent studies, we showed that EIF5A2 activates Rb phosphorylation by positively regulate CDKs. Phosphorylated Rb losses its function to inhibit E2F1 activity. As a member of the E2F transcription factor family, E2F1 participates in cell proliferation, differentiation and apoptosis and controls the cell cycle via a two-way regulatory mechanism. Playing a dual role in tumorigenesis, E2F1 is closely related to tumor progression and chemo-resistance [30, 31]. Furthermore, E2F1 also acts as a downstream target in regulating CSC properties in breast stem cell [32, 33]. In addition, the expression analysis from clinic specimens and TCGA database support the positive correlation between EIF5A2 and E2F1. In order to explore whether EIF5A2 regulated KLF4 in an E2F1-dependent manner, siRNA of E2F1 was conducted. The data showed that E2F1-KD inhibited the overexpression of KLF4 in EIF5A2-OE cells. Most interestingly, we found that E2F1 acts as an upstream transcription factor to activate KLF4 expression, witch participates in the EIF5A2-mediated modulation of CSC properties in EOC. Strikingly, KLF4 was previously reported to be as the direct downstream target in the E2F1-mediated pro-tumorigenic of melanoma [34]. The present study elucidates the previously unknown link between EIF5A2 and KLF4 and EIF5A2/pRb/E2F1/KLF4 axis is firstly confirmed which is a novel mechanism of the tumor initiation and chemo-resistance role of EIF5A2 and our results indicates that blocking this pathway may be a promising CSCs-specific therapeutic approach combined with platinum-based chemistry to treat EOC.

In our RNA-seq data, in addition to E2F1/KLF4 axis, multiple signaling pathways were downregulated and upregulated in EIF5A2-deficiency EOCs. Among these signaling pathways, Notch1/Hes1 which plays a crucial role in tumor development can be activated by EIF5A2. Thus, we inferred that Notch1/Hes1 might also be a downstream target of EIF5A2, but more studies are needed to investigate the mechanism. However, other altered pathways, such as PI3K-AKT, Hippo signaling pathways, which may also be involved in the maintenance of stemness modulated by EIF5A2. Whether these signals are regulated by EIF5A2 as well as play essential roles in EOC development awaits further investigation.

CSCs are not a fixed population, instead, with a cell trait of dramatic plasticity. One side of cancer stem cells is cellular senescence which is a stress-responsive cell-cycle arrest program. Maja Milanovic revealed that senescence-associated stemness exerts its detrimental, highly aggressive growth potential upon escape from cell-cycle blockade, which is critical for chemo-resistance and relapse [35]. Furthermore, CSCs can also be highly proliferative and they do not need to reside in a quiescent state as often assumed, which endows them with efficient tumorigenicity. It is known that cell cycle and division participate in the dramatic process. E2F1 is important for progression through the G1/S transition and controls the cell cycle via a two-way regulatory mechanism [36]. In addition to the role of cell cycle regulator, it also participates in the cell fate decisions of progenitor and stem cells [37]. KLF4 and Nanog were reported to be downstream target in the E2F1-mediated pro-tumorigenic of melanoma [34] and self-renewal of hepatocellular CSCs [38]. Interestingly, similar to E2F1, KLF4 also plays a dual role in tumorigenesis, which might be closely related with dramatic plasticity trait of stem cell. Further researches are needed to elucidate the suppose.

Consistent with these researches, we found that EIF5A2 is required to maintain KLF4 protein levels in our study, and E2F1 as an upstream transcriptional factor of KLF4 is activated by EIF5A2. KLF4 is an important mediator of pluripotent stemness which is associated with metastasis and chemo-resistance in cancer. There is an urgent need to find effective strategies to overcome drug resistance in the treatment of ovarian cancer. Our results suggest that reducing KLF4 expression by targeting the EIF5A2/E2F1/KLF4 axis combined with platinum-based chemistry may be a potential strategy.

## Conclusions

In conclusion, our study demonstrated the molecular mechanisms by which EIF5A2 regulates stem-like properties in EOC, further broadening the insight of the mechanisms regulating cancer stem cells and suggesting a promising CSCs-specific therapeutic target, EIF5A2, for future treatment of EOC.

## Supplementary Information

**Additional file 1: Table S1.** Sequences used in this study. **Table S2.** The percentages of ALDH+CD44+ cells in human ovarian cancer cell lines and MCAs samples from ascites. **Figure S1.** Isolation and verification of MCAs from ascites. Representative flow cytometry images of isolated MCAs from ascites. Subgroup of EpCAM+/CD45- were defined as epithelial tumor cells. **Figure S2.** Percentage of ALDH+ and CD44+ subset of MCAs cells. Flow cytometry analysis of ALDH and CD44 in single cells isolated from primary ovarian ascites. **Figure S3.** EIF5A2 expression and spheroid formation ability of three ovarian cancer cell lines. a. EIF5A2 protein expression of three ovarian cancer cell lines. b. EIF5A2 mRNA expression of three ovarian cancer cell lines. c. The number of spheroids formed from SKOV3, Hey, and HO-8910 cells was compared. **Figure S4.** EIF5A2 mRNA expression after knockdown in SKOV3 cells and overexpression in HO-8910 cells in different experiments. a. Spheroid formation of SKOV3 and HO-8910 cells, stem-related markers detection and immunofluorescent staining in SKOV3 derived spheroids. b. The proportion of stem cell analyzed by FCM. c. Drug sensitivity test. d. Subcutaneous tumorigenesis experiment. **Figure S5.** Quantitative analysis of Flow Cytometry. The proportion of CD44+/CD24− and CD133+ phenotype in SKOV3 spheroids and HO-8910 spheroids was analyzed by FCM. **Figure S6.** Quantitative analysis of spheroid formation ability. Single-cell suspensions with 3,000 cells were seeded in 6-well culture plates and cultured in semi-solid serum-free medium for 5 days. The number of spheroids formed was compared. **Figure S7.** Relative cell viability of SKOV3 / HO-8910 cells transfected with control siRNA or siEIF5A2 / vector or EIF5A2. CCK8 assay on SKOV3 and HO-8910 cells after incubation for 1, 3 and 5 days. **Figure S8.** Expression of CD133 and EIF5A2 in the subcutaneous tumors. Correlation analysis demonstrating that overexpression of EIF5A2 was positively correlated with CD133 expression ( r=0.7706, *p*<0.01). **Figure S9.** The mRNA levels of 7 genes enriched in the pathways regulating pluripotency of stem cell in SKOV3 cells with EIF5A2 knockdown were detected by qRT-PCR. **Figure S10.** Alteration of molecules in NOTCH pathway after EIF5A2 overexpression in HO-8910 cells.

## Data Availability

The datasets used and/or analyzed during the current study are available from the corresponding author on reasonable request.

## Abbreviations

OCSC: Ovarian cancer stem cells
SFM: Serum-free medium
RNA-seq: RNA-sequencing
EOC: Epithelial ovarian cancer
MCAs/MCSs: Multi-cellular aggregates/spheroids
EIF5A2: Eukaryotic initiation factor 5A2
IRS: Immunoreactive score
SI: Staining intensity
PP: Percentage of positive cells
qRT-PCR: Quantitative real-time polymerase chain reaction
KD: Knocked down
OE: Overexpressing
E2F1: E2F transcription factor 1
KLF4: Kruppel-like factor 4
OCT-4 (POU5F1): POU class 5 homeobox 1
ALDH1A1: Aldehyde dehydrogenase 1 family, member A1
Rb: Retinoblastoma
CDK: Cyclin-dependent kinase
PLK1: Polo-like kinase 1
CDC20: Cell division cycle 20
