# Supplementary material for: EIF5A2 enhances stemness of epithelial ovarian cancer cells via a E2F1/KLF4 axis
Source: Stem Cell Res Ther. 2021 Mar 16;12:186. doi: 10.1186/s13287-021-02256-2 (PMC7967996; doi:10.1186/s13287-021-02256-2)
Supplement: Supplementary file 1 — Additional file 1: Table S1. Sequences used in this study. Table S2. The percentages of ALDH+CD44+ cells in human ovarian cancer cell lines and MCAs samples from ascites. Figure S1. Isolation and verification of MCAs from ascites. Representative flow cytometry images of isolated MCAs from ascites. Subgroup of EpCAM+/CD45- were defined as epithelial tumor cells. Figure S2. Percentage of ALDH+ and CD44+ subset of MCAs cells. Flow cytometry analysis of ALDH and CD44 in single cells isolated from primary ovarian ascites. Figure S3. EIF5A2 expression and spheroid formation ability of three ovarian cancer cell lines. a. EIF5A2 protein expression of three ovarian cancer cell lines. b. EIF5A2 mRNA expression of three ovarian cancer cell lines. c. The number of spheroids formed from SKOV3, Hey, and HO-8910 cells was compared. Figure S4. EIF5A2 mRNA expression after knockdown in SKOV3 cells and overexpression in HO-8910 cells in different experiments. a. Spheroid formation of SKOV3 and HO-8910 cells, stem-related markers detection and immunofluorescent staining in SKOV3 derived spheroids. b. The proportion of stem cell analyzed by FCM. c. Drug sensitivity test. d. Subcutaneous tumorigenesis experiment. Figure S5. Quantitative analysis of Flow Cytometry. The proportion of CD44+/CD24− and CD133+ phenotype in SKOV3 spheroids and HO-8910 spheroids was analyzed by FCM. Figure S6. Quantitative analysis of spheroid formation ability. Single-cell suspensions with 3,000 cells were seeded in 6-well culture plates and cultured in semi-solid serum-free medium for 5 days. The number of spheroids formed was compared. Figure S7. Relative cell viability of SKOV3 / HO-8910 cells transfected with control siRNA or siEIF5A2 / vector or EIF5A2. CCK8 assay on SKOV3 and HO-8910 cells after incubation for 1, 3 and 5 days. Figure S8. Expression of CD133 and EIF5A2 in the subcutaneous tumors. Correlation analysis demonstrating that overexpression of EIF5A2 was positively correlated with CD133 expressi [file 13287_2021_2256_MOESM1_ESM.pdf]

Supplemental Materials

Table S1 Sequences used in this study

Primers for reverse transcriptase polymerase chain reaction	
EIF5A2	Forward:5'-GGCTTCCAGCACTTACCCTA-3'
	Reverse:5'-ATGGTCGTCCTTTGAGCACC-3'
KLF4	Forward:5'-CATCTCAAGGCACACCTGCGAA-3'
	Reverse:5'-TCGGTCGCATTTTTGGCACTGG-3'
E2F1	Forward:5'-GGACCTGGAAACTGACCATCAG-3'
	Reverse:5'-CAGTGAGGTCTCATAGCGTGAC-3'
CDC20	Forward:5'-CGGAAGACCTGCCGTTACATTC-3'
	Reverse:5'-CAGAGCTTGCACTCCACAGGTA-3'
PLK1	Forward:5'-GCACAGTGTCAATGCCTCCAAG-3'
	Reverse:5'-GCCGTACTTGTCCGAATAGTCC-3'
CDC45	Forward:5'-TGGATGCTGTCCAAGGACCTGA-3'
	Reverse:5'-CAGGACACCAACATCAGTCACG-3'
YWHAH	Forward:5'-ACGACATGGCCTCCGCTATGAA-3'
	Reverse:5'-GCTAATGACCCTCCAGGAAGATC-3'
PCNA	Forward:5'-CAAGTAATGTCGATAAAGAGGAGG-3'
	Reverse:5'-GTGTCACCGTTGAAGAGAGTGG-3'
CCNB1	Forward:5'-GACCTGTGTCAGGCTTTCTCTG-3'
	Reverse:5'-GGTATTTTGGTCTGACTGCTTGC-3'
SKP2	Forward:5'-GATGTGACTGGTCGGTTGCTGT-3'
	Reverse:5'-GAGTTCGATAGGTCCATGTGCTG-3'
BUB1B	Forward:5'-GTGGAAGAGACTGCACAACAGC-3'
	Reverse:5'-TCAGACGCTTGCTGATGGCTCT-3'
ACVR1	Forward:5'-GACGTGGAGTATGGCACTATCG-3'
	Reverse:5'-CACTCCAACAGTGTAATCTGGCG-3'
GLI3	Forward:5'-TCAGCAAGTGGCTCCTATGGTC-3'
	Reverse:5'-GCTCTGTTGTCGGCTTAGGATC-3'
ID1	Forward:5'-GTTGGAGCTGAACTCGGAATCC-3'
	Reverse:5'-ACACAAGATGCGATCGTCCGCA-3'
HES1	Forward:5'-GGAAATGACAGTGAAGCACCTCC-3'
	Reverse:5'-GAAGCGGGTCACCTCGTTCATG-3'
FZD4	Forward:5'-TTCACACCGCTCATCCAGTACG-3'

	Reverse:5'-ACGGGTTCACAGCGTCTCTTGA-3'
PAX6	Forward:5'-CTGAGGAATCAGAGAAGACAGGC-3'
	Reverse:5'-ATGGAGCCAGATGTGAAGGAGG-3'
Sequences used for siRNAs (5'to 3')	
EIF5A2	GGAUCUUAAACUGCCAGAATT
	UUCUGGCAGUUUAAGAUCCTT
E2F1	GACCACCUGAUGAAUAUCUTT
	AGAUAUUCAUCAGGUGGUCTT
KLF4	CCAUUAUCAAGAGCUCAUGCCACCG
	CGGUGGCAUGAGCUCUUGAUAAUGG
Lentivirus target sequence	
EIF5A2	GGAUCUUAAACUGCCAGAATT
NC	TTCTCCGAACGTGTCACG

Table S2 The percentages of ALDH+CD44+ cells in human ovarian cancer cell lines and MCAs samples from ascites

Cell lines or MCAs samples	Percentage (%)
SKOV3	3.8±0.1
HO-8910	2.5±0.05
HEY	2.7±0.04
patients #1	8.1±0.1
patients #2	24.5±0.5
patients #3	32.2±0.6
patients #4	3.9±0.2
patients #5	17.5±0.4
patients #6	8.9±0.5
patients #7	15.6±0.8
patients #8	1.4±0.2
patients #9	35.6±0.4
patients #10	13.4±0.6
patients #11	2.2±0.08
patients #12	23.6±0.15

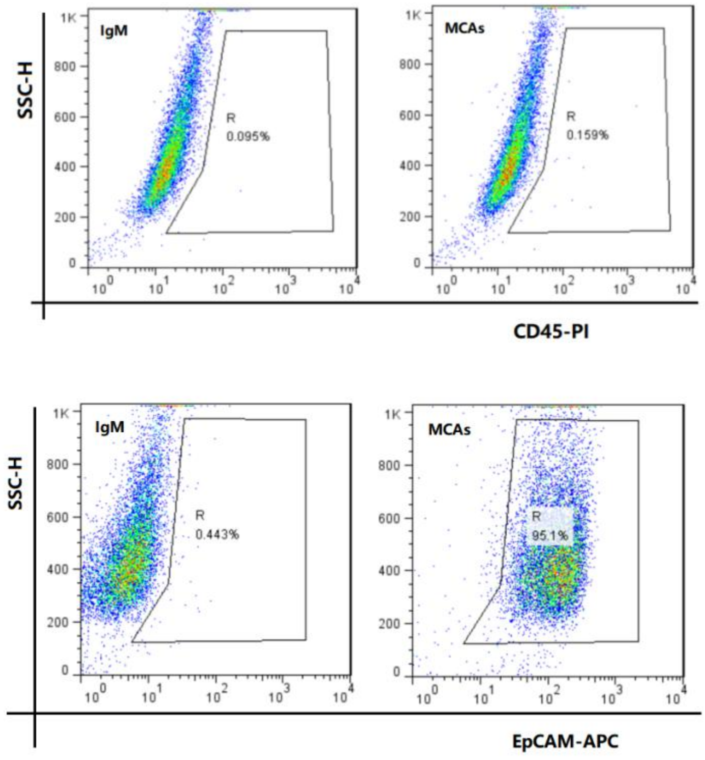

Figure S1 Isolation and verification of MCAs from ascites

Representative flow cytometry images of isolated MCAs from ascites. Subgroup of EpCAM+/CD45- were defined as epithelial tumor cells.

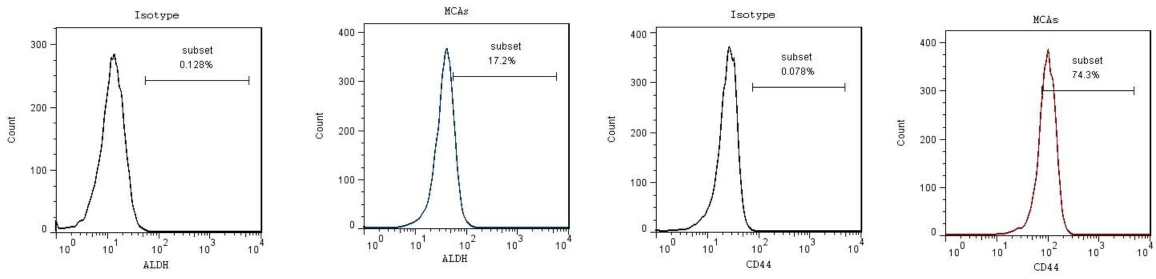

Figure S2 Percentage of ALDH+ and CD44+ subset of MCAs cells

Flow cytometry analysis of ALDH and CD44 in single cells isolated from primary ovarian ascites.

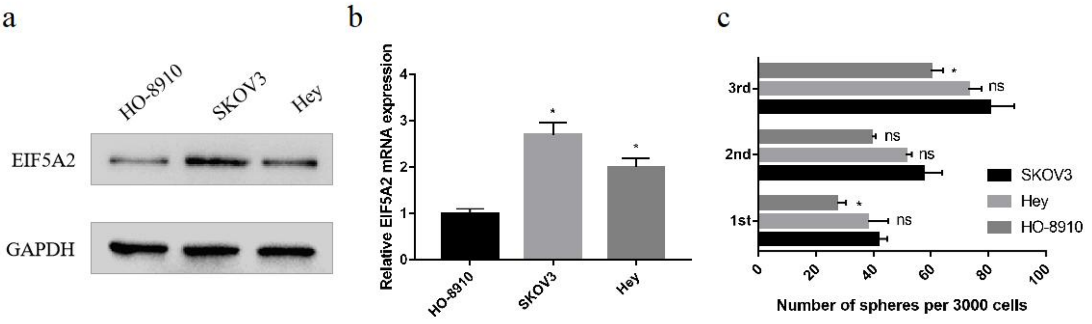

Figure S3 EIF5A2 expression and spheroid formation ability of three ovarian cancer cell lines. a. EIF5A2 protein expression of three ovarian cancer cell lines. b. EIF5A2 mRNA expression of three ovarian cancer cell lines. c. The number of spheroids formed from SKOV3, Hey, and HO-8910 cells was compared.

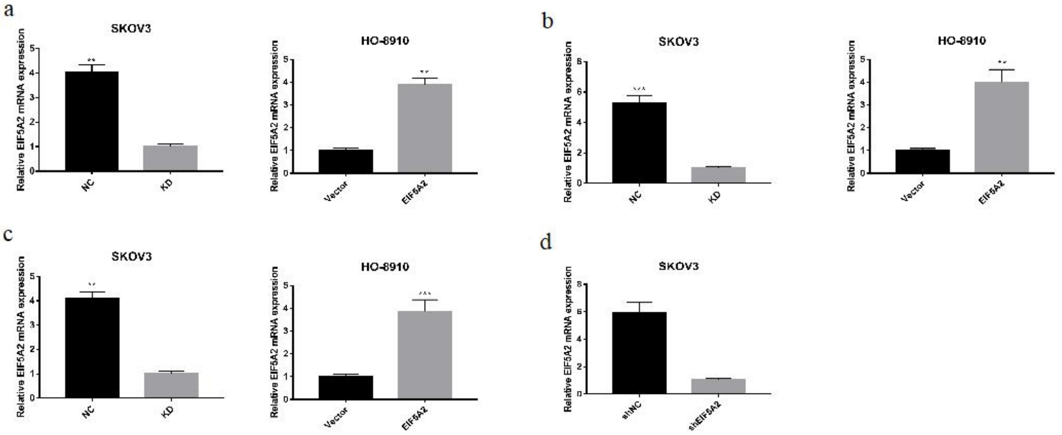

Figure S4 EIF5A2 mRNA expression after knockdown in SKOV3 cells and overexpression in HO-8910 cells in different experiments. a. Spheroid formation of SKOV3 and HO-8910 cells, stem-related markers detection and immunofluorescent staining in SKOV3 derived spheroids. b. The proportion of stem cell analyzed by FCM. c. Drug sensitivity test. d. Subcutaneous tumorigenesis experiment.

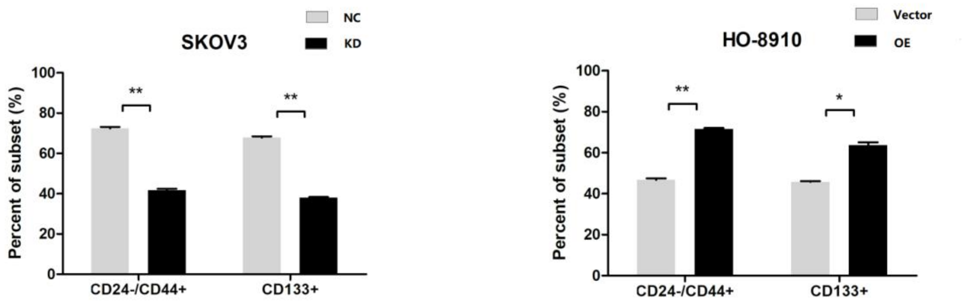

Figure S5 Quantitative analysis of Flow Cytometry

The proportion of CD44+/CD24- and CD133+ phenotype in SKOV3 spheroids and HO-8910 spheroids was analyzed by FCM.

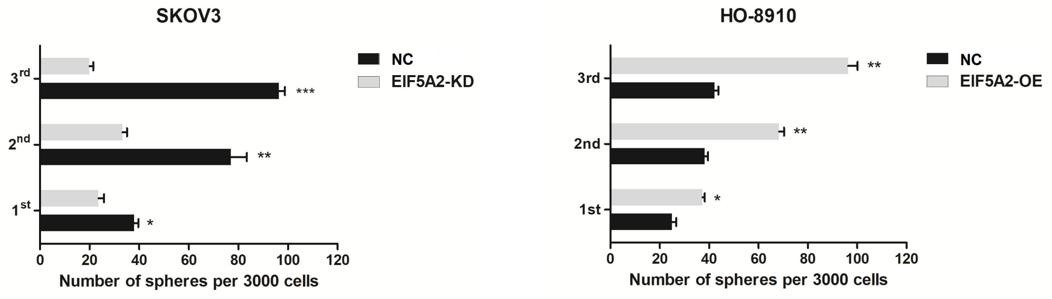

Figure S6 Quantitative analysis of spheroid formation ability

Single-cell suspensions with 3,000 cells were seeded in 6-well culture plates and cultured in semi-solid serum-free medium for 5 days. The number of spheroids formed was compared.

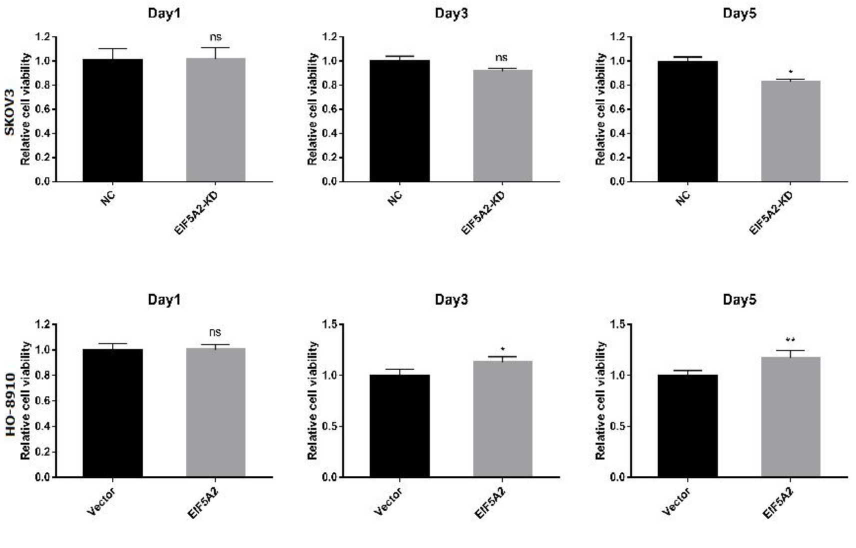

Figure S7 Relative cell viability of SKOV3 / HO-8910 cells transfected with control siRNA or siEIF5A2 / vector or EIF5A2. CCK8 assay on SKOV3 and HO-8910 cells after incubation for 1, 3 and 5 days.

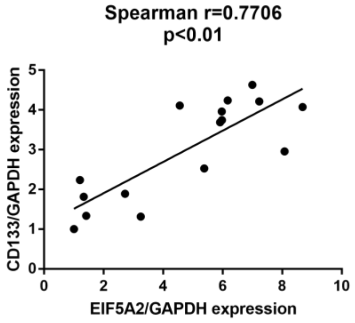

Figure S8 Expression of CD133 and EIF5A2 in the subcutaneous tumors.

Correlation analysis demonstrating that overexpression of EIF5A2 was positively correlated with CD133 expression ($r=0.7706$, $p<0.01$)

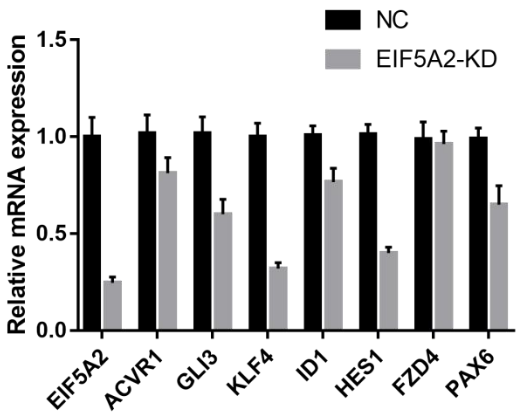

Figure S9 The mRNA levels of 7 genes enriched in the pathways regulating pluripotency of stem cell in SKOV3 cells with EIF5A2 knockdown were detected by qRT-PCR.

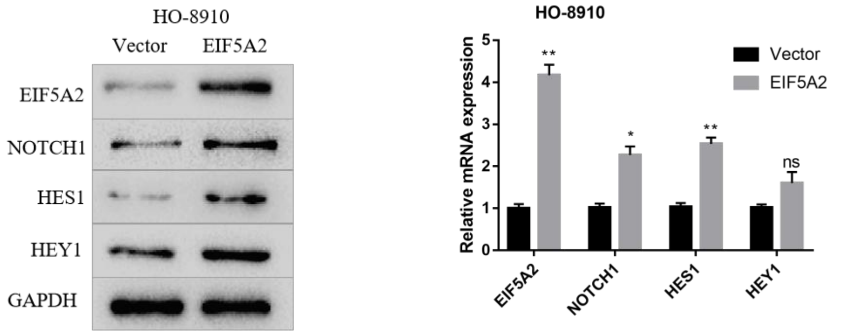

Figure S10 Alteration of molecules in NOTCH pathway after EIF5A2 overexpression in HO-8910 cells.
